# Application of fractal dimension combined with radiomics and clinical scores in recurrence prediction of atrial fibrillation after radiofrequency ablation

**DOI:** 10.3389/fcvm.2026.1730263

**Published:** 2026-07-22

**Authors:** Chenhao Li, Sixiang Jia, Yang Yang, Qingru Zhu, Shudong Xia

**Affiliations:** Department of Cardiology, the Fourth Affiliated Hospital of School of Medicine, and International School of Medicine, Zhejiang University, Yiwu, China

**Keywords:** atrial fibrillation (AF), fractal dimension (FD), radiofrequency catheter ablation (RFCA), radiomics, recurrence prediction

## Abstract

**Background:**

Atrial fibrillation (AF) is the most common clinical arrhythmia. Radiofrequency catheter ablation (RFCA) is a primary treatment, but its success rate (50%–80%) varies by AF type and individual differences, making recurrence a key clinical challenge. Previous recurrence assessments ignored atrial remodeling-related complex structural features; fractal dimension (FD) quantifies structural complexity, and radiomics enables high-throughput feature extraction. Integrating these with clinical scores to build a composite model may improve risk stratification accuracy.

**Methods:**

This single-center retrospective study enrolled 536 AF patients who underwent RFCA between January 2016 and March 2024, with follow-up through March 2025. Preoperative cardiac computed tomography venography (CTV) images and clinical data, such as APPLE and CHA₂DS₂-VASc scores, were collected via the Hospital Information System (HIS). Automated segmentation of the left atrium (LA) and left atrial appendage (LAA) was performed using specialized software, from which radiomics features were extracted and the FD was calculated. Patients were stratified and randomly divided into a training set (*n* = 375) and a validation set (*n* = 161) in a 7:3 ratio. Following feature selection, a multidimensional recurrence prediction model was constructed, and its parameters were optimized using five-fold cross-validation.

**Results:**

Among the 536 patients, AF recurrence occurred in 139 cases (25.9%). Specifically, recurrence was observed in 111 cases (25.9%) in the training set and 28 cases (25.9%) in the test set. The integrated model, which combined the FD, radiomics features from both the LA and the LAA, as well as the APPLE and CHA₂DS₂-VASc scores, achieved an area under the curve (AUC) of 0.87 in the validation set. Its performance was significantly superior to that of models based solely on clinical scores (APPLE score AUC = 0.57; CHA₂DS₂-VASc score AUC = 0.66) or radiomics features alone (LAA model AUC = 0.77; LA model AUC = 0.62).

**Conclusion:**

The integrated model incorporating multidimensional features enables effective and accurate prediction of AF recurrence after ablation, providing a practical tool for clinical individualized management. Its utility requires further validation and broader application through prospective studies.

## 1 Introduction

Atrial fibrillation (AF) is the most common clinical arrhythmia, with its core mechanism being the suppression of sinoatrial node rhythm by abnormal electrical activity within the atria, leading to atrial rhythm disorders (1). Epidemiological data demonstrate that the global prevalence of AF increased from 33.5 million in 2010 to 59 million in 2019 (2), and it is anticipated to continue rising significantly in the future (3). Currently, catheter-based radiofrequency ablation (RFCA) centered on pulmonary vein isolation serves as the primary treatment strategy for AF (4). However, influenced by AF type and individual patient characteristics, the postoperative success rate ranges only from 50% to 80%, making recurrence a critical unresolved clinical issue (5). Left atrial enlargement and dysfunction represent the core pathophysiological changes of AF. Previous studies have confirmed that left atrial morphology and function, as well as left atrial appendage (LAA) morphology, are closely associated with the risk of postoperative recurrence (6). Although cardiac computed tomography venography (CTV), routinely performed preoperatively to rule out intracardiac thrombi, can clearly display the anatomical relationship between the left atrium (LA) and pulmonary veins as well as the detailed morphology of the LAA, its clinical application largely relies on visual qualitative analysis or simple quantitative indices (7–11), which limits its ability to accurately extract recurrence-related structural features.

Radiomics enables the objective characterization of tissue heterogeneity through high-throughput extraction of quantitative features, such as intensity and texture (12, 13). It has been successfully applied in cardiovascular research for predicting myocardial infarction and differentiating myocardial hypertrophy (14, 15), indicating its potential for AF recurrence prediction. Nevertheless, its capacity to quantify complex atrial and appendicular structures associated with AF recurrence—such as the irregular texture of fibrotic areas or subtle density variations caused by intracavitary stasis—remains limited (16). As a high-order feature focusing on quantifying structural complexity, fractal dimension (FD) can convert irregular morphologies into numerical indices, thereby complementing the evaluation dimensions of traditional radiomics (17). While FD has been applied in cardiovascular medicine (e.g., distinguishing athlete's heart from hypertrophic cardiomyopathy (17)), its application in CTV images of patients undergoing AF ablation remains unexplored. In addition, existing predictive tools for postoperative AF recurrence, such as the APPLE score, have obvious limitations due to their failure to incorporate microstructural features, making it difficult to comprehensively capture factors related to AF recurrence.

Therefore, this study aims to extract traditional radiomics features and the FD from pre-procedural CTV images of AF patients undergoing RFCA. By integrating these with the APPLE and CHA₂DS₂-VASc scores to incorporate clinical risk factors, we will construct and validate a multimodal predictive model for AF recurrence. The objective is to enhance the identification accuracy of patients at high risk of postoperative recurrence and provide a tool for individualized clinical management.

## 2 Method

**Figure 1:**
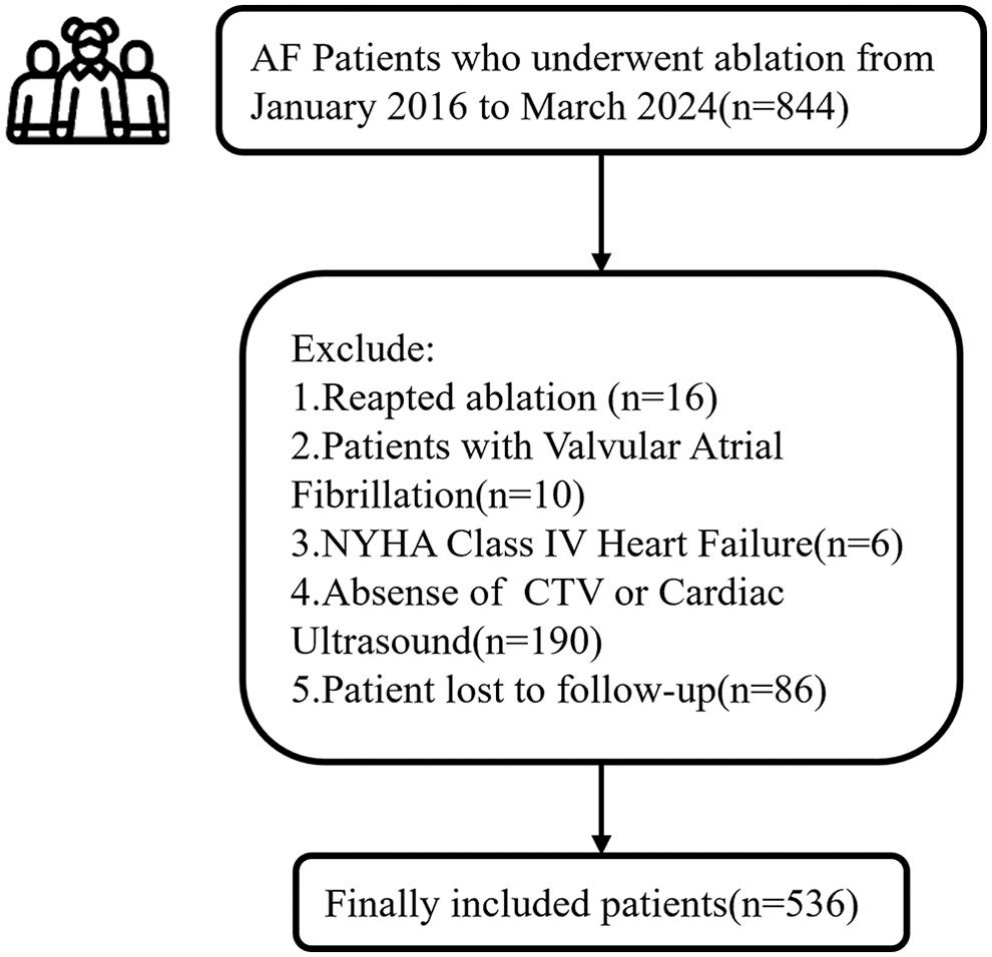
Inclusion and exclusion flow diagram.

This study utilized retrospective data from a single center. Patients diagnosed with AF who underwent RFCA between January 2016 and March 2024 were identified via the Hospital Information System (HIS). Demographic information, traditional AF recurrence scores (Supplementary A4, APPLE and CHA2DS2-VASc (18)) and computed tomography (CT) data were exported from the system. The primary endpoint was AF recurrence, with follow-up completed through March 2025. Details of the ablation procedure and postoperative follow-up are provided in Supplementary A1, A2. The patient selection process is illustrated in Figure 1. Based on this process, a total of 536 patients were finally enrolled. This study was approved by the Ethics Committee of The Fourth Affiliated Hospital of Zhejiang University School of Medicine (Approval No. K2025254) and conducted in accordance with the Declaration of Helsinki. As this was a retrospective study and postoperative follow-up was part of routine clinical care, the requirement for informed consent was waived. All analyses and manuscript reporting were completed in full compliance with each checklist item of TRIPOD + AI (19).

### 2.1 Definition of AF recurrence

According to the latest ACC/AHA guidelines, AF recurrence is defined as the occurrence of atrial arrhythmias (atrial tachycardia, atrial flutter, or AF) lasting longer than 30 s following the procedure. Recurrences occurring within 3 months postoperatively are classified as early recurrences, while those occurring after 3 months are considered late recurrences (20). Outcome assessors were blinded to patients' preoperative imaging features and clinical scores.

### 2.2 CT protocols

We used a standardized CT scanning protocol to ensure consistent cardiac image acquisition across patients, minimizing measurement errors from rhythm variations. Further details on the CT acquisition are provided in Supplementary A3. Prior to analysis, the images underwent preprocessing, which included resampling voxel sizes and discretizing Hounsfield units. Voxel dimensions were resampled to 3 × 3 × 3 mm (in the x-, y-, and z-axes, respectively) to correct for voxel resolution variations from acquisition. To improve the visualization of the LAA and reduce interference from surrounding organs, the window width was set to 400, and the window level to 40 during CT scans.

### 2.3 Regions of interest segmentation

This paper implements segmentation of the LA and LAA, with all steps performed on the MMIS V1.3.1 platform. Specifically, deep learning-based organ segmentation is performed on the CT images using a model integrated within MMIS, which is based on TotalSegmentator (21), to obtain the initial segmentation results of the LA and LAA. Subsequently, further morphological processing and regional connectivity analysis are applied to optimize the boundaries and remove artifacts. Finally, spatial relationship analysis is conducted between the LAA segmentation results and the LA segmentation results to ensure that their boundaries are accurate and well-separated, thereby obtaining the final segmentation results of the LA and LAA (21, 22).Three-dimensional reconstructions of the segmented LA and LAA from multiple viewing angles are provided in the Supplementary Material.

### 2.4 Extraction of radiomics and FD features

The open-source package Pyradiomics (http://pypi.org/project/pyradiomics/) was used to extract hand-crafted radiomic (HCR) features from the three-dimensional region of interest (3D ROI) of the segmented LA and LAA. A total of 1,037 features were obtained for each structure (LA and LAA), including 107 original features, 186 Gaussian-filtered features, and 744 wavelet-filtered features, the distribution chart of each feature's proportion is shown in Supplementary Figure S8.

This study utilized Python 3.10 to perform FD analysis on the LA and LAA for a systematic assessment of their structural characteristics. The analysis proceeded along two dimensions: firstly, the morphological FD, calculated from the segmentation masks (binary images with the target region as 1 and background as 0) using the box-counting method to quantify the spatial complexity of the structural contours; and secondly, the texture FD, derived by applying the differential box-counting method to grayscale images (where Hounsfield Unit (HU) values reflect tissue density) that combined HU values with the segmentation masks, thereby characterizing the irregularity of internal tissue density distribution. The results for both FD types are presented in box plots illustrating their overall distributions, where a higher FD indicates greater structural complexity. Detailed procedures for FD calculation and fitting are provided in the Supplementary A5.

### 2.5 AF recurrence model development and validation

Patients were first divided into a training set and a test set using stratified random sampling in a 7:3 ratio. The total sample size and outcome events met the events per variable (EPV) principle for machine learning prediction model development and internal validation. For the training set, feature selection was performed using Python based on radiomics features extracted from the LA and LAA, as well as FD features. The process involved: initially screening features significantly associated with the outcome using the *F*-test; subsequently filtering out highly correlated features using Pearson correlation coefficient (threshold: 0.95); and finally retaining features with absolute coefficients >1e-4 via Lasso regression.

Based on the above selection results, we constructed 13 single-module batch machine learning classification models using Python. The input features were respectively: LA radiomics features, LAA radiomics features, FD features, APPLE score, and CHA₂DS₂-VASc score. The model types included Logistic Regression (LR), Support Vector Machine (SVM, with linear/poly/sigmoid kernels), K-Nearest Neighbors (KNN), Random Forest (RF), Extremely Randomized Trees (ExtraTree), Extreme Gradient Boosting (XGBoost), Multi-Layer Perceptron (MLP), and Decision Tree (DT).

To construct the integrated model, we first calculated weights based on the Area Under the Curve (AUC) performance of the optimal single-module models on the test set. Subsequently, using the predicted probabilities output by the top five optimal single-module models as input features, we retrained the models using the same set of machine learning algorithms mentioned above, ultimately building the integrated model.

To mitigate the risk of overfitting, an independent hyperparameter grid was utilized for each module, optimized through five-fold cross-validation. Key metrics, including the AUC and accuracy, along with their 95% confidence intervals (CIs), were calculated based on 100 bootstrap samples. Receiver Operating Characteristic (ROC) curves for both the training and test sets were plotted to identify the optimal integrated model. Key predictive factors for each model were identified through feature importance analysis. Decision Curve Analysis (DCA), implemented in Python, was employed to compare the clinical utility of the integrated model against single-module models. Patients were stratified into five risk quintiles according to predicted probabilities of the final integrated model. The number and proportion of recurrent events were calculated under two analytical definitions: recurrence including early relapse and late recurrence only. Subgroup analyses, stratified by age, sex, and AF type, were conducted to evaluate model robustness, with comparative AUC results presented in a forest plot. A sensitivity analysis was performed to assess the impact of early recurrence on model performance. The composition of feature categories was visualized using a circular pie chart, and the risk of overfitting was assessed by the difference in AUC between the training and test sets.

### 2.6 Statistical analysis

**Figure 2:**
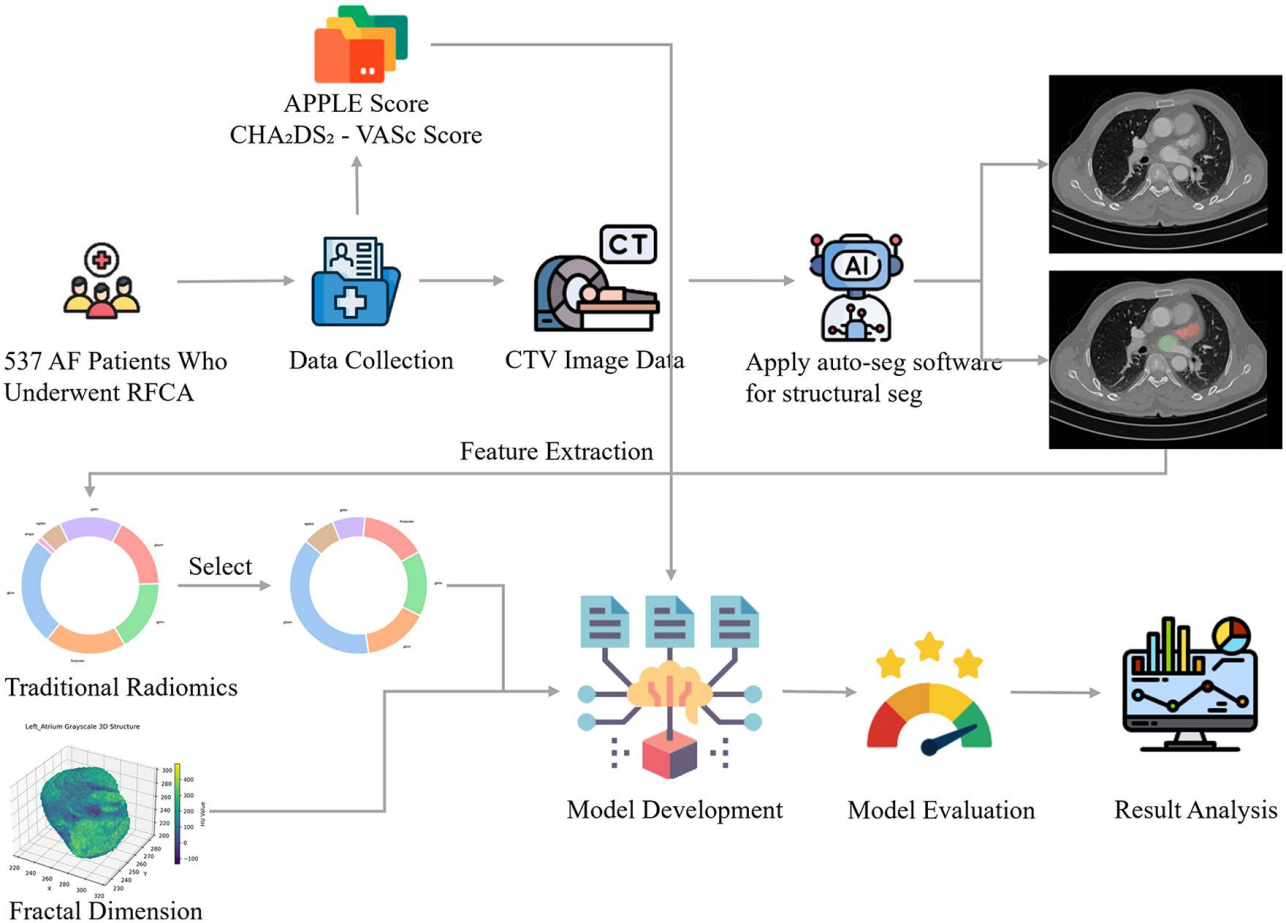
Study flow chart of procedures.

Continuous variables are presented as mean ± standard deviation, and categorical variables as counts (n) and percentages (%). Student's t-test was used for normally distributed quantitative data, while Levene's test assessed homogeneity of variances. The Mann–Whitney *U*-test was applied for non-normally distributed data. Chi-square tests compared categorical data. All tests were two-tailed, with statistical significance set at *P* < 0.05. Statistical analyses were conducted using R (version 4.0.2; https://www.r-project.org) and Python (version 3.10; https://www.python.org). Figure 2 shows the study flowchart.

## 3 Result

### 3.1 Characteristics of the datasets

A total of 536 patients with AF who underwent RFCA were enrolled based on pre-defined inclusion and exclusion criteria. The cohort comprised 338 males (63%), with a median age of 68 years and a median follow-up duration of 2.24 years. During the follow-up period, 139 patients experienced AF recurrence, corresponding to a recurrence rate of 25.03%.

Using stratified random sampling, patients were divided into training and test cohorts in a 7:3 ratio, resulting in 428 patients assigned to the training cohort and 108 to the test cohort. In the training cohort, 111 patients (25.9%) experienced recurrence, while 28 patients (25.9%) had AF recurrence in the test cohort. The baseline characteristics of the patients are detailed in Table 1.

**Table 1:** Characteristics of the training and validation cohorts.

Variable	Total	Group		*P* value
		Train	Test	
N	536	428	108	
Age (years)	68.00 (61.00–72.00)	68.00 (60.00–72.00)	67.00 (61.00–72.25)	0.949
AF Duration (months)	3.00 (0.33–24.00)	3.00 (0.33–24.00)	3.00 (0.50–24.00)	0.965
CHA2DS2-VASc Score	2.00 (1.00–3.00)	2.00 (1.00–3.00)	2.00 (1.00–3.00)	0.245
Left Atrial Diameter (mm)	37.00 (33.00–41.32)	37.00 (33.00–42.00)	37.00 (33.30–40.50)	0.887
LVEF (%)	64.00 (59.30–68.35)	64.00 (59.27–68.00)	63.50 (59.88–69.28)	0.333
Creatinine (*μ*mol/L)	72.00 (62.00–86.00)	71.50 (62.00–86.00)	74.50 (62.00–88.00)	0.483
eGFR (mL/min/1.73 m²)	91.00 (77.00–103.16)	92.00 (77.80–103.16)	87.00 (76.00–102.50)	0.369
LA Morphological FD	1.89 (1.81–1.95)	1.89 (1.81–1.95)	1.88 (1.81–1.95)	0.663
LAA Morphological FD	2.26 (2.20–2.32)	2.26 (2.20–2.31)	2.249 (2.19–2.34)	0.911
LA Textural FD	2.38 (2.31–2.45)	2.38 (2.31–2.45)	2.38 (2.32–2.47)	0.819
LAA Textural FD	2.81 (2.73–2.88)	2.81 (2.73–2.88)	2.79 (2.73–2.87)	0.557
Follow-up Time (years)	2.24 (1.44–3.52)	2.30 (1.43–3.60)	2.12 (1.50–3.36)	0.963
APPLE Score	1.00 (0.00–2.00)	1.00 (0.75–2.00)	1.00 (0.00–2.00)	0.087
Gender				0.93
Female	198 (36.9%)	159 (37.1%)	39 (36.1%)	
Male	338 (63.1%)	269 (62.9%)	69 (63.9%)	
Hypertension				0.898
No	213 (39.7%)	169 (39.5%)	44 (40.7%)	
Yes	323 (60.3%)	259 (60.5%)	64 (59.3%)	
Coronary Heart Disease				0.18
No	424 (79.1%)	333 (77.8%)	91 (84.3%)	
Yes	112 (20.9%)	95 (22.2%)	17 (15.7%)	
Diabetes				0.531
No	438 (81.7%)	347 (81.1%)	91 (84.3%)	
Yes	98 (18.3%)	81 (18.9%)	17 (15.7%)	
Type of AF				0.23
Paroxysmal AF	333 (62.1%)	260 (60.7%)	73 (67.6%)	
Persistent AF	203 (37.9%)	168 (39.3%)	35 (32.4%)	
AF Recurrence				0.167
Early Recurrence	34 (6.3%)	31 (7.2%)	3 (2.8%)	
Late Recurrence	105 (19.6%)	80 (18.7%)	25 (23.1%)	
None Recurrence	397 (74.1%)	317 (74.1%)	80 (74.1%)	

Data are presented as mean (SD) or median (interquartile range) for continuous variables and *n* (%) for categorical variables.

### 3.2 Module contribution weights & corresponding features

LAA module: Following feature selection, 19 features were retained and entered into candidate machine learning models. Based on the AUC values in the training set, the random forest (RF) algorithm was selected as the optimal model (AUC = 0.859, Table 2). The feature importance ranking and the distribution of radiomics feature categories before and after selection are displayed in Supplementary Figures S8–S9.

**Table 2:** Summary of predictive indicators for AF recurrence.

Model Name	Dataset Type	ACC	AUC	AUC_95%CI	Sensitivity	Specificity	PPV	NPV	F1
Left Atrium (linear_SVM)	Train	0.624	0.685	0.633–0.733	0.755	0.578	0.390	0.870	0.513
Left Atrium (linear_SVM)	Test	0.643	0.626	0.459–0.755	0.663	0.633	0.430	0.850	0.487
Left Atrium Appendage (RF)	Train	0.763	0.859	0.818–0.898	0.810	0.747	0.543	0.920	0.642
Left Atrium Appendage (RF)	Test	0.761	0.777	0.666–0.877	0.756	0.762	0.538	0.899	0.621
Fractal Dimension (DT)	Train	0.705	0.813	0.773–0.845	0.781	0.678	0.471	0.906	0.578
Fractal Dimension (DT)	Test	0.749	0.735	0.641–0.841	0.701	0.766	0.521	0.880	0.590
APPLE (LR)	Train	0.619	0.618	0.556–0.676	0.566	0.638	0.359	0.809	0.434
APPLE (LR)	Test	0.676	0.571	0.459–0.670	0.423	0.768	0.435	0.789	0.389
CHA2DS2-VASc (poly_SVM)	Train	0.439	0.485	0.417–0.543	0.683	0.352	0.274	0.812	0.356
CHA2DS2-VASc (poly_SVM)	Test	0.552	0.660	0.551–0.741	0.867	0.439	0.361	0.907	0.503
Fusion model (poly_SVM)	Train	0.807	0.910	0.885–0.933	0.907	0.771	0.585	0.960	0.709
Fusion model (poly_SVM)	Test	0.820	0.869	0.776–0.941	0.816	0.822	0.632	0.928	0.702

LA Module: Thirteen features were retained after feature selection. After being incorporated into candidate models, the linear support vector machine (Linear_SVM) was identified as the optimal model according to the training set AUC values (AUC = 0.685, Table 2). The corresponding feature importance ranking and radiomics feature category distribution before and after selection are shown in Supplementary Figures S8–S9.

FD Module: As shown in Supplementary Figure S10, there were significant positive correlations between morphological FD and textural FD for both the LA and LAA, with data points forming obvious clusters in the two-dimensional space, suggesting substantial differences in fractal characteristics between the two structures. Supplementary Figure S11 further quantified the distribution of FD values in the two structures. The median values of both morphological FD and textural FD were significantly higher in the LA than in the LAA. After feature selection, LA textural FD and LAA morphological FD were included in the FD-based prediction model. Following model training and comparison, the decision tree (DT) algorithm (AUC = 0.813, Table 2) was selected as the optimal model, and the feature importance score of LA textural FD was significantly higher than that of LAA morphological FD (Supplementary Figure S12, 0.7 vs. 0.3).

APPLE Score and CHA₂DS₂-VASc Score Modules: As no feature selection was required, the score values were directly input into the aforementioned 13 candidate machine learning models. The Logistic Regression (LR) model (AUC: 0.617) and the Polynomial Support Vector Machine (Poly_SVM) model (AUC: 0.485) were ultimately selected as the optimal models for the APPLE score and CHA₂DS₂-VASc score modules, respectively.

### 3.3 Comparison of AF recurrence prediction models

The above five sub-models were integrated to construct a combined prediction model for AF recurrence after RFCA. Based on the aforementioned 13 machine learning algorithms, model training and optimization were performed on the training set using five-fold cross-validation. Among these, the polynomial kernel support vector machine (poly_SVM) achieved the best performance and was thus determined as the final combined model, in which the FD module showed the highest feature importance (Supplementary Figure S13).

**Figure 3:**
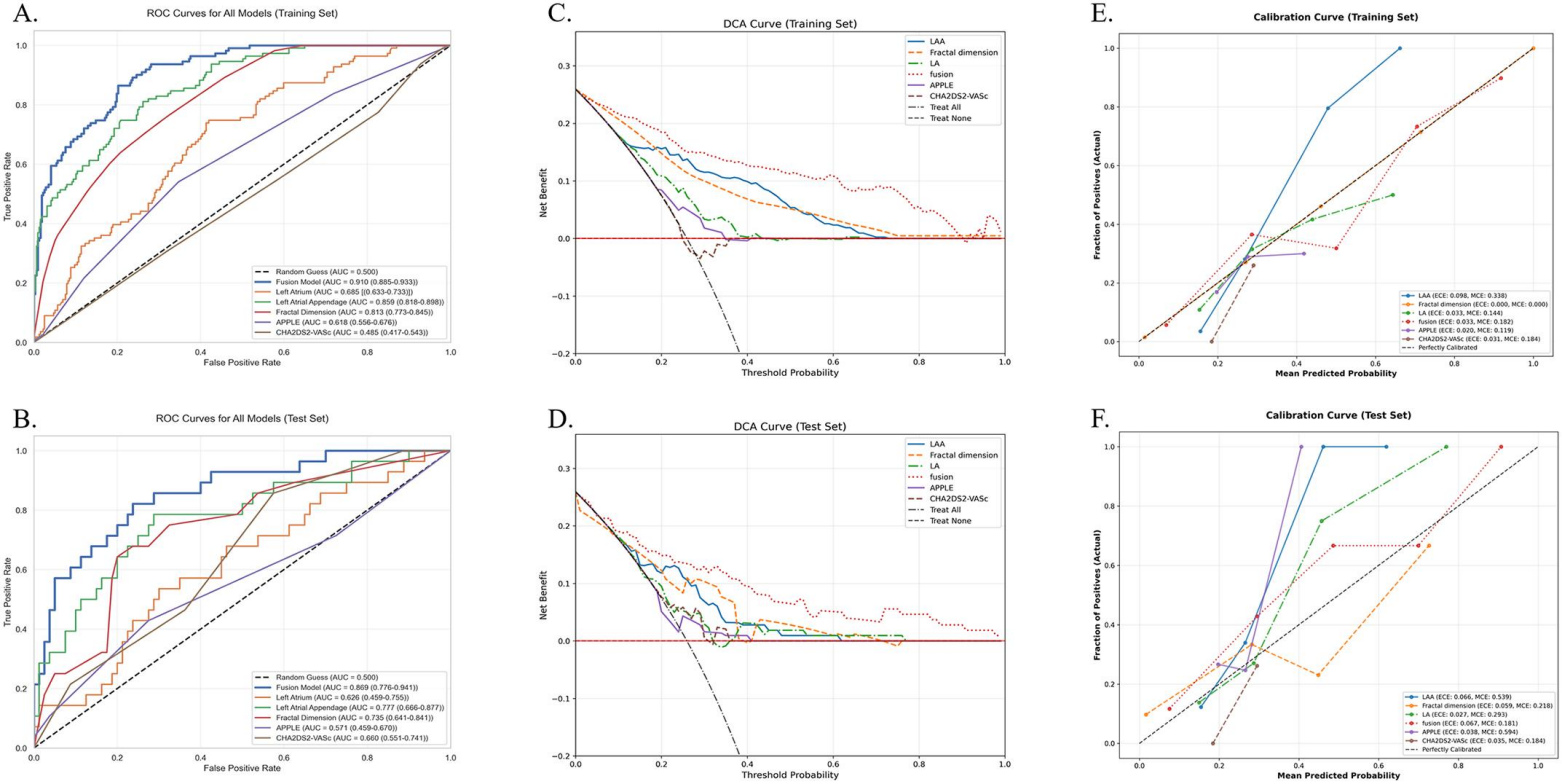
Comparison of AF recurrence prediction models. **(A)** ROC curves of various models for predicting AF recurrence in the training cohort. **(B)** DCA Curves of various models for predicting AF recurrence in the training cohort. **(C)** Calibration curves of various models for predicting AF recurrence in the training cohort. **(D)** ROC curves of various models for predicting AF recurrence in the test cohort. **(E)** DCA curves of various models for predicting AF recurrence in the test cohort. **(F)** Calibration curves of various models for predicting AF recurrence in the test cohort.

As shown in Figure 3, the fusion model achieved the optimal discriminative performance in the ROC curves of both the training and test sets, with an AUC of 0.87 (95% CI: 0.78–0.94, Table 2) in the test set. The model exhibited consistent performance between the training and test sets, with no obvious overfitting. DCA demonstrated that the net benefit of the fusion model was consistently higher than that of the “treat all” and “treat none” strategies across a wide range of threshold probabilities, indicating favorable clinical utility. Calibration curves and quantitative indices revealed that the LA model achieved the lowest expected calibration error (ECE = 0.027) and the best calibration performance. Although the fusion model had a slightly higher ECE (0.067), its maximum calibration error (MCE = 0.181) was well controlled, indicating robust overall calibration. Across five risk quintiles stratified by predicted probabilities (Supplementary Table S1), recurrence rates rose generally with elevated risk scores under both early-recurrence-included and early-recurrence-excluded definitions.

### 3.4 Model robustness assessment

**Figure 4:**
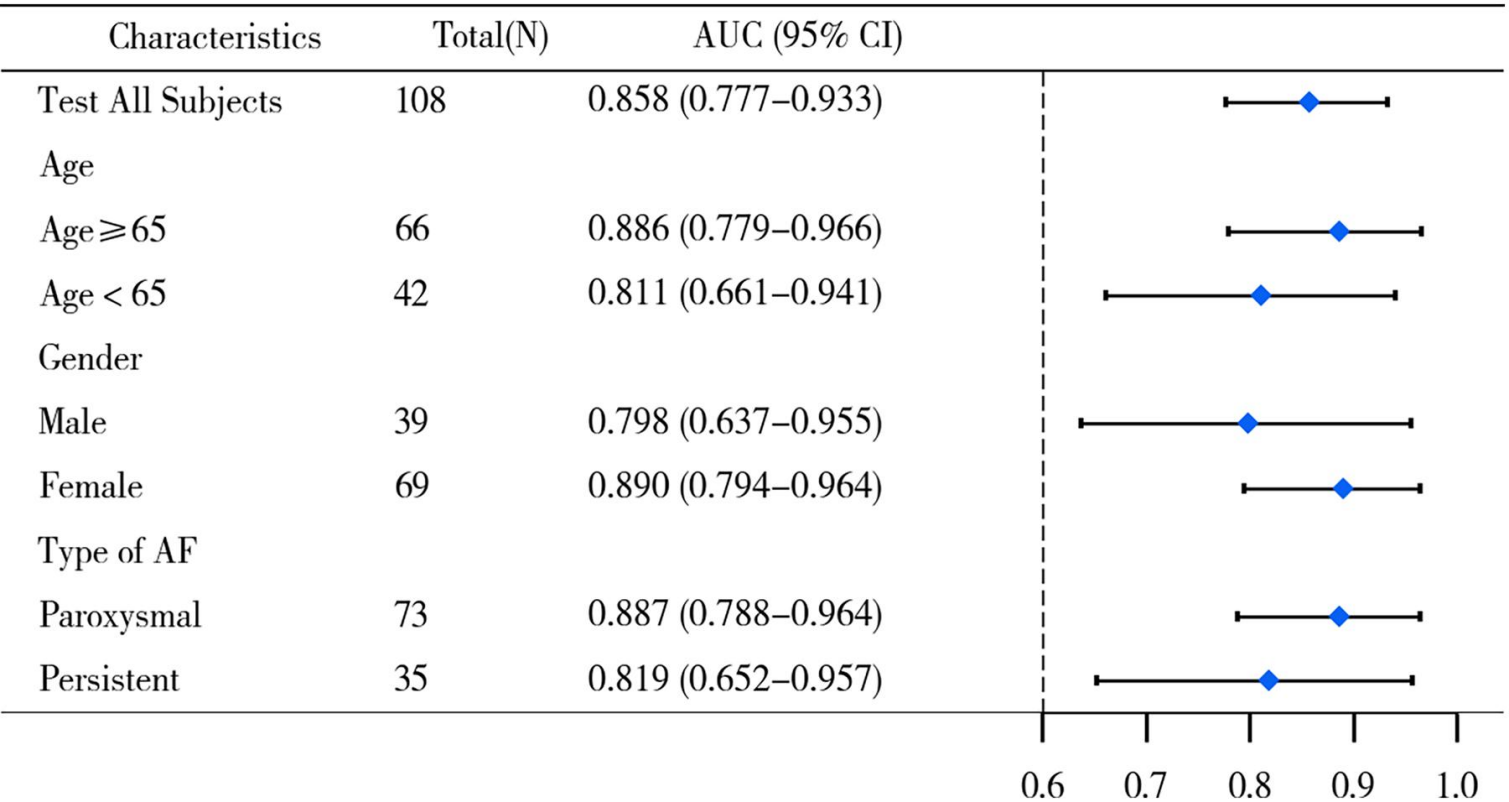
Subgroup analysis of the combined model in the test set.

To assess the robustness of the combined model, we conducted subgroup analyses in the test set (*n* =108) stratified by sex, age (cutoff, 65 years) and type of AF. The AUC values for these subgroups were calculated and visualized in a forest plot (Figure 4). The result showed no significant heterogeneity in predictive performance, indicating a consistent discriminative ability across the patient populations.

**Figure 5:**
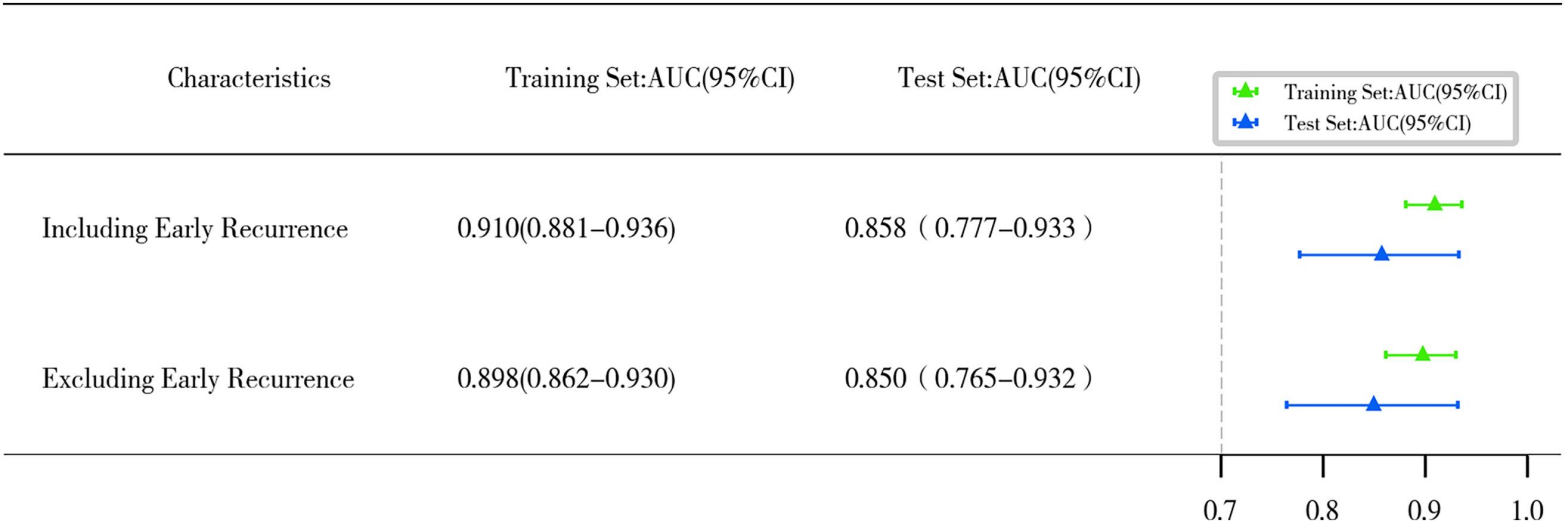
Sensitivity analysis: CIs of model performance metrics with inclusion and exclusion of early AF recurrence events.

Sensitivity analysis was performed to evaluate the model's stability with and without early AF recurrence. The results demonstrated that the CIs of the model performance metrics remained stable (Figure 5).

## 4 Discussion

This study integrated left atrial and left atrial appendage imaging features, including radiomic signatures and fractal dimension, combined with two well-recognized clinical scores (CHA₂DS₂-VASc and APPLE), to construct and validate a predictive model for recurrence after radiofrequency catheter ablation of atrial fibrillation. Current risk scores for post-ablation atrial fibrillation recurrence have limited overall discriminative efficacy. Multiple head-to-head comparative studies have demonstrated that conventional thromboembolic scores (CHA₂DS₂-VASc, CHADS₂) and dedicated AF recurrence scores (APPLE, BASE-AF2, CAAP-AF, MB-LATER, DR-FLASH, HATCH, etc.) yield modest predictive performance, with AUC values consistently ranging from 0.545 to 0.669 (23). The predictive efficacy of CHA₂DS₂-VASc and APPLE observed in our cohort is consistent with these external findings, further confirming the inherent limitations of single conventional scoring systems. In contrast, the integrated model established in the present study achieves an AUC of 0.87, exhibiting significantly better discriminative ability and superior clinical risk stratification value compared with all existing traditional risk scores.

FD as an advanced imaging metric focusing on “quantifying complex structures,” differs from radiomics, which involves high-throughput extraction of basic structural features like intensity and texture. It specifically targets “complex structures that are difficult to quantify visually.” In this study, the single-module model using FD alone achieved an AUC of 0.813, and it was assigned a high weight in the fused model (Supplementary Figure S13), confirming its role as a key predictive feature for AF recurrence. Notably, the model ultimately selected two indicators: LA textural FD and LAA morphological FD. The weight of LA textural FD in the model was significantly higher than that of LAA morphological FD, a finding highly consistent with the quantitative capability of FD for complex structures and the pathophysiology of AF.

From the essence of FD, its core lies in quantifying the “self-similarity” and “geometric complexity” of irregular structures through numerical values (24). Textural FD, based on tissue CT values (HU values), focuses on structural heterogeneity (such as the degree of fibrosis). In contrast, morphological FD reflects contour complexity, leaning more towards “adaptive changes in external morphology.” This offers a significant complementary advantage to traditional radiomics. Traditional radiomics provides substantial data support for structural assessment by extracting basic structural information like intensity and texture features, focusing on tissue density differences and texture patterns (25). FD, utilizing methods like box-counting and differential box-counting, transforms “visually difficult-to-quantify irregularity” into objective numerical values. Here, LA textural FD quantifies the heterogeneity of density distribution within the atrial tissue, closely related to atrial structural remodeling induced by AF (26). LAA morphological FD quantifies morphological changes in the LAA caused by AF (27). These two aspects complement each other from the dimensions of “internal tissue structural heterogeneity of the LA” and “external spatial morphological characteristics of the LAA,” jointly providing a basis for recurrence risk assessment.

Within the FD model, the higher weight of LA textural FD compared to LAA morphological FD reflects their differing core contributions and intrinsic relevance to the AF pathophysiological process, demonstrating the ability of FD to characterize the disease essence. From anatomical and electrophysiological perspectives, the LAA is generally less associated with the etiology of AF initiation, but repeated AF episodes can lead to anatomical and physiological remodeling of the LAA (28). The initiation and maintenance of AF typically require electrophysiological and anatomical substrates within the atrium, which include changes at the cellular or molecular level (e.g., tissue fibrosis, altered ion channel properties) and the organ level (e.g., atrial dilation) (28). Existing studies have established left atrial fibrosis and left atrial dilation as recognized risk factors for AF recurrence after ablation (29, 30). AF leads to disorganization of atrial myocardial cells and fibrosis, while atrial fibrosis can disrupt normal electrical conduction, forming reentrant circuits, thereby significantly increasing AF susceptibility (31). LA textural FD can indirectly reflect this pathological process through textural complexity; fibrosis causes a mixed distribution of healthy and diseased tissue on CT images, increasing LA textural FD due to enhanced textural self-similarity. The elevated FD indirectly reflects fibrosis and also indicates the resulting electrical conduction heterogeneity and disordered electrical activity, making it a “core signal” for recurrence risk. This association mechanism, where fibrosis causes mixed tissue distribution quantified by FD, has been validated in other disease areas, such as rectal cancer, where intratumoral fibrosis leading to mixed tissue distribution was correlated with increased FD (32).

The LAA, as an appendage of the LA, undergoes morphological changes primarily as a secondary response to atrial pathology: specifically, left atrial remodeling due to AF can cause dilation of the LAA (including its orifice) (33); furthermore, increased left atrial pressure resulting from electrical remodeling and fibrosis can lead to decreased LAA contractile function by increasing its afterload (34). Studies have shown correlations between various LAA imaging parameters (e.g., LAA morphology, orifice area, length, and diameter) and AF recurrence (35). However, these secondary changes related to LAA morphology, while capturable by morphological FD and useful for assisting in predicting local thrombotic risk (complex morphology predisposing to blood stasis) (36), do not reflect global determinants of recurrence like atrial electrical remodeling and overall fibrosis. Therefore, they serve merely as “ancillary signals,” with lower weight than LA textural FD.

In recent years, studies have attempted to predict AF recurrence using radiomics, providing an important approach for non-invasive assessment of recurrence risk. This study is also conducted within the radiomics technical framework, consistent with this core direction. Previous research often focused on “macro-morphological features” (e.g., number of LAA lobes, orifice diameter) (37) or “conventional texture features” (e.g., gray-level co-occurrence matrix) (38). The core logic involves quantifying the association between atrial structural changes and recurrence risk through imaging features, which aligns with this study's core idea of “using imaging features to reflect atrial remodeling for predicting recurrence.” For instance, the I-SCORE model proposed by Liu et al., built using left atrial segmentation combined with clinical factors, shares a consistent assessment logic regarding the association between atrial anatomical structure and recurrence risk with this study's focus on atrial structural features. However, it is important to note limitations of previous studies: on one hand, they often did not reach the level of microstructural quantitative analysis, failing to further explore the deep pathological significance of structural heterogeneity; on the other hand, models like Liu et al.'s did not incorporate LAA morphological features and did not involve FD for quantifying microstructure (39). In contrast, while continuing the consistent rationale of “quantifying atrial structure using radiomics,” this study further incorporates LA textural FD, LAA morphological FD, and radiomic features. This both fills the gap in microstructural quantification and better aligns with the pathological understanding that “global LA remodeling is core, LAA morphological change is ancillary” (28, 40), constituting an improvement and extension of previous research.

This model enables precise risk stratification for patients after pulmonary vein isolation and optimizes the current uniform postoperative follow-up strategy. For patients at low recurrence risk, follow-up intervals can be appropriately extended and redundant examinations reduced; high-risk individuals receive intensified cardiac rhythm surveillance and early intervention. This model thus provides a quantitative tool for refined, individualized post-ablation management. In parallel, pulsed field ablation (PFA) is gradually replacing radiofrequency ablation as the primary procedure for *de novo* atrial fibrillation owing to its myocardial tissue selectivity. Most existing predictive tools rely on thermal injury parameters specific to radiofrequency ablation and cannot be readily applied to the PFA workflow. By contrast, our model is constructed based on intrinsic atrial remodeling signatures without incorporating radiofrequency thermal injury metrics. It can therefore be extended to evaluate recurrence risk after PFA, matching the evolving landscape of atrial fibrillation ablation techniques.

### 4.1 Limitations

Although this study developed and validated a multi-dimensional prediction model for AF recurrence after radiofrequency catheter ablation, which outperformed traditional scoring systems, several limitations warrant consideration and improvement in future investigations.

First, as a single-center retrospective study, selection bias could not be entirely eliminated. More importantly, the model was only validated internally, without external validation using an independent cohort, which restricts its generalizability. Caution should be taken not to overinterpret its predictive performance or extrapolate its clinical utility. In addition, data were obtained from the HIS, some of which lacked standardized documentation, and the sample size of 537 cases was relatively modest compared with multi-center studies. Further prospective, multi-center studies with rigorous external validation are therefore warranted to improve the reliability and generalizability of the findings.

Second, the study enrolled patients undergoing ablation between 2016 and 2024, during which considerable advances occurred in ablation techniques and clinical strategies. For instance, high-power ablation protocols such as 50W and 90W have gradually emerged and been widely adopted, accompanied by ongoing refinements in catheter technology and procedural workflows. Such improvements may enhance treatment efficacy and reduce overall recurrence rates, thereby altering the distribution of outcome events in this study. Because the model was constructed across an era of evolving ablation practice, its predictive performance and feature-outcome relationships may have been influenced by historical improvements in therapeutic effectiveness. Further validation is thus needed to confirm its applicability under contemporary standardized ablation practice.

Finally, although all patients completed at least 1 year of follow-up, which is sufficient for assessing 1-year recurrence, only those enrolled before March 2023 had reached a follow-up duration of 2 years or longer, limiting the sample size available for long-term analysis. This heterogeneity in follow-up duration hampers the evaluation of the model's long-term stability after ablation. Extended follow-up will be required once all patients complete at least 2 years of follow-up to assess the long-term predictive value of the model.

## 5 Conclusion

We developed and validated a multi-dimensional feature-integrated recurrence prediction model incorporating FD, radiomics, and clinical scores. This model can reliably predict the treatment outcome of radiofrequency catheter ablation for AF.

## Data Availability

The raw data supporting the conclusions of this article will be made available by the authors, without undue reservation.
