# Supplementary material for: Application of fractal dimension combined with radiomics and clinical scores in recurrence prediction of atrial fibrillation after radiofrequency ablation
Source: Front Cardiovasc Med. 2026 Jul 22;13:1730263. doi: 10.3389/fcvm.2026.1730263 (PMC13437442; doi:10.3389/fcvm.2026.1730263)
Supplement: Supplementary file 1 [file Supplementaryfile1.docx]

**Supplementary Methods**

Figure S1. Three-dimensional reconstructions of the segmented left atrium (LA) and left atrial appendage (LAA) from multiple viewing angles

Supplementary A1. Radiofrequency ablation procedure for atrial fibrillation.

Supplementary A2. Postablation follow-up of atrial fibrillation.

Supplementary A3. Contrast enhanced CT scan annotation.

Supplementary A4. Calculation of APPLE and CHA2DS2-VASc Scores.

Supplementary A5. Fractal Dimension (FD) Calculation.

Supplementary A6. Representative Fractal Dimension Maps of Left Atrium and Left Atrial Appendage in Patients with and without Atrial Fibrillation Recurrence.

Figure S8. Proportion of Major Radiomics Feature Categories in Left Atrium and Left Atrial Appendage Before and After Feature Selection.

Figure S9. Feature Importance Ranking of Radiomics Models for Left Atrium and Left Atrial Appendage.

Figure S10. Correlation between Morphological and Textural Fractal Dimensions in the Left Atrium and Left Atrial Appendage.

Figure S11. Distribution of Morphological and Textural Fractal Dimensions in the Left Atrium and Left Atrial Appendage.

Figure S12. Feature Importance for Left Cardiac Structure Based on Fractal Dimension.

Figure S13. Learning Curve and Sub-model Weights of the poly_SVM Model in the Training Set.

Table S1. Recurrence events and recurrence rates across five risk quintiles of the integrated prediction model

**Figure S1. Three-dimensional reconstructions of the segmented left atrium (LA) and left atrial appendage (LAA) from multiple viewing angles**

Supplementary A1. Radiofrequency Catheter Ablation Procedure for Atrial Fibrillation

The Radiofrequecy Catheter Ablation (RFCA) procedure was conducted under sedation, with continuous monitoring of ECG and oxygen saturation throughout the ablation process. Transesophageal cardiac ultrasound was carried out 24 h before RFCA to rule out the presence of left atrial thrombus. Standardized circumflex pulmonary vein isolation was performed on all patients. Intraoperatively, a sheath was inserted through the femoral vein, and after accessing the left atrium via septal puncture, the Carto 3 three-dimensional anatomical mapping system was utilized for precise localization during circumflex pulmonary vein isolation. Ablation power ranged from 35-45 W for the anterior wall and 30-40 W for the posterior wall. Additional ablation pathways, such as superior vena cava isolation, roof line or mitral isthmus line ablation of the left atrium, and tricuspid isthmus ablation, were determined by the operator based on individual requirements. Pulmonary venous potentials were recorded using a LASSO® NAV catheter before, during, and after the ablation procedure to assess its effectiveness. The ablation endpoint was achieved when the bi-directional transmission (afferent and efferent) of pulmonary venous potentials ceased completely for at least 30 min. In cases of persistent atrial fibrillation or other atrial tachycardia post-ablation, direct current cardioversion could be considered as an option to restore sinus rhythm.

**Supplementary A2. Postablation follow-up of atrial fibrillation**

Following ablation, all patients undergo outpatient follow-up and 24-hour ambulatory electrocardiogram monitoring at 1, 3, and 6 months post-procedure. At 12 months post-procedure, outpatient follow-up and 7-day long-term ambulatory electrocardiogram monitoring are conducted. Subsequently, outpatient follow-up and 24-hour ambulatory electrocardiogram monitoring are performed every 6 months.

Sequential oral anticoagulant therapy should be initiated the day after RFCA to minimize the interruption of anticoagulation treatment. According to individual CHA2DS2-VASc scores, AF patients are advised to continue oral anticoagulants for a minimum of two months. Persistent AF patients are prescribed amiodarone for three months post-RFCA to uphold sinus rhythm and undergo regular monitoring for medication side effects. If there is no recurrence during the three-month blanking period, patients with paroxysmal AF should refrain from using class I or III antiarrhythmic drugs. In the event of AF recurrence, β-receptor blockers are prioritized as the first-line rate control treatment, with propafenone being considered if symptoms persist despite β-receptor blocker usage. All class I or III antiarrhythmic drugs are ceased at the conclusion of the blanking period.

**Supplementary A3. Contrast enhanced CT scan annotation.**

A 256-slice spiral CT scan was performed to conduct left atrial pulmonary vein CTA in atrial fibrillation patients prior to ablation. For patients with arrhythmia or instability, metoprolol was administered for control. The scanning range extended from 0.5 cm below the tracheal bifurcation to the cardiac diaphragm. Iopamidol contrast agent (350 mg I/mL, 0.8 mL/kg) was injected into the antecubital vein using a high-pressure injector at a flow rate of 4–5 mL/s. Scanning parameters included wide body axis mode, collimation of 256 × 0.625 mm, rotation time of 0.28 s/r, tube voltage of 80–100 kV, tube current of 280–1200 mAs, and a matrix size of 512 × 512.

**Supplementary A4. Calculation of APPLE and** **CHA2DS2-VASc Scores**

The APPLE total score ranges from 0 to 5 points, with the following specific criteria: Age over 65 years contributes 1 point; persistent atrial fibrillation adds 1 point; impaired estimated glomerular filtration rate (eGFR) [< 60 mL/(min. 1.73 m2)], adds 1 point; left atrial diameter ≥ 4.3 cm contributes 1 point; left ventricular ejection fraction (LVEF) <50% adds 1 point.

The CHA2DS2-VASc score is utilized to estimate the risk of stroke in patients with atrial fibrillation (AF). The score is calculated by assigning points for the presence of specific risk factors: Congestive Heart Failure/Left Ventricular Dysfunction: 1 point, Hypertension: 1 point, Age 75 years or older: 2 points, Diabetes Mellitus: 1 point, Stroke/Transient Ischemic Attack (TIA)/Thromboembolism: 2 points, Vascular Disease (e.g., prior myocardial infarction, peripheral artery disease, aortic plaque): 1 point,Age 65-74 years: 1 point.

**Supplementary A5. Fractal Dimension (FD) Calculation.**

In this study, morphological fractal dimensions (based on binary masks) and textural fractal dimensions (based on CT gray-scale images) were computed for the left atrium (LA) and left atrial appendage (LAA). All FD calculations were performed on three-dimensional (3D) volumetric data using the box-counting method and its modified version, the differential box-counting method.

1. Morphological Fractal Dimension Calculation

Morphological FD values of the LA and LAA were calculated from their binary segmentation masks using a 3D box-counting algorithm. The binary mask was used as input, where a voxel value of 1 indicated the target anatomical region and 0 represented the background. The box size ranged from a minimum of 2 voxels to a maximum of one‑fourth the smallest dimension of the input 3D image. A sequence of 10 initial box sizes was generated on a logarithmic base‑2 scale within this range, and duplicate values were removed to form a unique scale sequence. Each box in the 3D volume was traversed, and boxes containing at least one non-zero voxel were counted. The valid box sizes were transformed to log(1/ε), where ε denotes the box scale, and the corresponding counts were transformed to log(N(ε)), where N represents the number of boxes covering the target region. A linear regression was then applied, and the slope of the fitted line was defined as the morphological FD. The coefficient of determination (R²) was calculated to evaluate the goodness of fit, and only results with at least three valid box counts were retained to ensure data quality.

2. Textural Fractal Dimension Calculation

Textural FD values of the LA and LAA were derived from CT gray-scale volumetric data (Hounsfield Units, HU) within the masked regions using a 3D differential box-counting method, a modified box-counting approach for gray-scale images. Voxels outside the mask were set to NaN to restrict the analysis to the region of interest. The box size range and scale generation rules were identical to those used for morphological FD calculation: a minimum of 2 voxels, a maximum of one‑fourth the smallest dimension of the 3D image, and 10 unique box sizes derived from a logarithmic base‑2 scale. For each box in the 3D volume, the minimum and maximum HU values within the box were computed. If the maximum HU value exceeded the minimum, the number of boxes required to cover the gray-level range was determined using np.ceil((box_max − box_min) / box_size). If no gray-level variation was observed within the box, a count of 1 was assigned. Boxes containing only NaN values were skipped. Textural FD was obtained from the linear regression of log(1/ε) against log(N(ε)), and only results with at least three valid box counts were preserved. No resampling, interpolation, normalization, or filtering was applied to the HU values, and the spatial resolution of the mask and CT images remained consistent throughout processing.

3. Key Parameters and Computational Dimensionality

Both morphological and textural FD calculations were performed directly on 3D volumetric data across the x, y, and z axes, without separate analysis of individual 2D slices, to preserve the three-dimensional anatomical characteristics of cardiac structures. For illustrative purposes, if the input 3D image dimensions were (256, 256, 128), the box sizes ranged from 2 to 64 voxels (128//4), yielding a unique scale sequence of [2, 4, 8, 16, 32, 64] after deduplication. All computational parameters were set according to established empirical conventions in radiomic fractal analysis, and result reliability was ensured by filtering based on goodness of fit (R²) and valid box counts.

4. Visualization and Validation

For the first 10 patients, log–log plots of box-counting regression were generated with log(1/ε) on the x-axis and log(N(ε)) on the y-axis, and the FD value and R² were annotated to validate fitting performance. Three-dimensional reconstruction models and representative 2D slices were also visualized to confirm the accuracy of the regions used for FD calculation.

**Supplementary A6. Representative Fractal Dimension Maps of Left Atrium and Left Atrial Appendage in Patients with and without Atrial Fibrillation Recurrence**

This supplementary presents representative three-dimensional (3D) reconstruction models and fractal dimension (FD) maps of the left atrium (LA) and left atrial appendage (LAA) in two typical patients, including one with atrial fibrillation (AF) recurrence and one without AF recurrence, to intuitively display the differences of morphological and textural FD between the two groups.

Patient with AF recurrence: Figure S2 shows the 3D reconstruction model of LA and LAA based on CT Hounsfield Unit (HU) values, clearly displaying the anatomical structure and spatial distribution of LA and LAA. Figure S3 presents the morphological FD maps of LA and LAA, reflecting the complexity of the anatomical contour; the higher morphological FD values indicate that the boundary of LA and LAA is more irregular. Figure S4 shows the textural FD maps of LA and LAA, which characterize the spatial heterogeneity of CT density (HU values) within the LA and LAA region; the higher textural FD values suggest a more complex and inhomogeneous distribution of internal CT density.

Patient without AF recurrence: Figure S5 shows the 3D reconstruction model of LA and LAA based on CT HU values, with a relatively regular anatomical structure compared with the patient with AF recurrence. Figure S6 presents the morphological FD maps of LA and LAA, with lower morphological FD values, indicating that the contours of LA and LAA are smoother and more regular. Figure S7 shows the textural FD maps of LA and LAA, with lower textural FD values, suggesting a more homogeneous and uniform distribution of internal CT density.

Figure S2 3D reconstruction model of the left atrium (LA) and left atrial appendage (LAA) based on CT HU values in a patient with atrial fibrillation (AF) recurrence

Figure S3 Morphological fractal dimension (FD) maps of the LA and LAA in a patient with AF recurrence

Figure S4 Texture fractal dimension (FD) maps of the LA and LAA in a patient with AF recurrence

Figure S5 3D reconstruction model of the LA and LAA based on CT HU values in a patient without AF recurrence

Figure S6 Morphological fractal dimension (FD) maps of the LA and LAA in a patient without AF recurrence

Figure S7 Texture fractal dimension (FD) maps of the LA and LAA in a patient without AF recurrence

**Figure S8. Proportion of Major Radiomics Feature Categories in Left Atrium and Left Atrial Appendage Before and After Feature Selection.**

(A) Left Atrium (LA) before and after selection. (B) Left Atrial Appendage (LAA) before and after selection.

**Figure S9. Feature Importance Ranking of Radiomics Models for Left Atrium and Left Atrial Appendage.**

(A) Left Atrium (LA) Feature Importance Ranking. (B) Left Atrial Appendage (LAA) Feature Importance Ranking.

**Figure S10. Correlation between Morphological and Textural Fractal Dimensions in the Left Atrium and Left Atrial Appendage.**

**Figure S11. Distribution of Morphological and Textural Fractal Dimensions in the Left Atrium and Left Atrial Appendage.**

**Figure S12. Feature Importance for Left Cardiac Structure Based on Fractal Dimension.**

**Figure S13. Learning Curve and Sub-model Weights of the poly_SVM Model in the Training Set.**

(A) Learning Curve of the poly_SVM Model in the Training Set. (B) Weight Distribution of Each Sub-model.

**Table S1. Recurrence events and recurrence rates across five risk quintiles of the integrated prediction model**

| Risk Quintile | Total Patients | Total Recurrence (Including Early Recurrence) | Recurrence (Excluding Early Recurrence) | Total Recurrence Rate (Including Early Recurrence, %) | Recurrence Rate (Excluding Early Recurrence, %) |
| --- | --- | --- | --- | --- | --- |
| Quintile 1 | 23 | 2 | 1 | 8.7 | 4.35 |
| Quintile 2 | 15 | 2 | 2 | 13.33 | 13.33 |
| Quintile 3 | 28 | 11 | 11 | 39.29 | 39.29 |
| Quintile 4 | 29 | 7 | 6 | 24.14 | 20.69 |
| Quintile 5 | 13 | 6 | 5 | 46.15 | 38.46 |
